# Probing the phenomenon of trained immunity in invertebrates during a transgenerational study, using brine shrimp *Artemia* as a model system

**DOI:** 10.1038/srep21166

**Published:** 2016-02-15

**Authors:** Parisa Norouzitallab, Kartik Baruah, Priyanka Biswas, Daisy Vanrompay, Peter Bossier

**Affiliations:** 1 Laboratory of Aquaculture & Artemia Reference Center, Department of Animal Production, Faculty of Bioscience Engineering, Ghent University, Rozier 44, Ghent 9000, Belgium; 2 Lab of Immunology and Animal Biotechnology, Faculty of Bioscience Engineering, Ghent University, Coupure 653, Ghent 9000, Belgium

## Abstract

The invertebrate’s innate immune system was reported to show some form of adaptive features, termed trained immunity. However, the memory characteristics of innate immune system and the mechanisms behind such phenomena remain unclear. Using the invertebrate model *Artemia*, we verified the possibility or impossibility of trained immunity, examining the presence or absence of enduring memory against homologous and heterologous antigens (*Vibrio* spp.) during a transgenerational study. We also determined the mechanisms behind such phenomenon. Our results showed the occurrence of memory and partial discrimination in *Artemia’s* immune system, as manifested by increased resistance, for three successive generations, of the progenies of *Vibrio*-exposed ancestors towards a homologous bacterial strain, rather than to a heterologous strain. This increased resistance phenotype was associated with elevated levels of *hsp70* and *hmgb1* signaling molecules and alteration in the expression of key innate immunity-related genes. Our results also showed stochastic pattern in the acetylation and methylation levels of H4 and H3K4me3 histones, respectively, in the progenies whose ancestors were challenged. Overall results suggest that innate immune responses in invertebrates have the capacity to be trained, and epigenetic reprogramming of (selected) innate immune effectors is likely to have central place in the mechanisms leading to trained immunity.

Evidences collected to date demonstrated that invertebrates do not possess a specific immune system similar to that of vertebrates. In fact, they lack lymphocytes and functional immunoglobulin, the key hallmarks of the vertebrate adaptive immune system^1^, and hence are thought to lack both adaptivity (the ability to respond rapidly upon re-exposure to a particular pathogen) and specific memory^2^,^3^. With only innate immunity, invertebrates are believed to act naively to each new encounter with pathogens^4^. However, a few studies on insects as model organisms indicated that the invertebrate innate immune system could display characteristics of immunological memory, which are functionally equivalent to adaptive immunity in vertebrates^2^,^5^. These claims for memory-like immunity in invertebrates, termed as trained immunity, were based largely on observations in only a few insect species and a limited selection of read-outs, such as improved immune responses or increased resistance towards microbial infections following prior exposure to pathogens and/or immune elicitors^2^,^6^. In those studies, the effects of innate immune memory appeared to persist for days, weeks, almost the lifetime of the adult, or sometimes across generation i.e., the protective/immune responses were transmitted from parents to offspring^7^,^8^. Despite these (phenomenological) observations, the ‘phenomena of trained immunity’ in invertebrates still remains a topic of debate^9^. It is because most of the earlier studies addressing trained immunity in invertebrates failed to make direct assessment of the immunological parameters and correlates it to the functional responses (i.e., resistance towards homologous or heterologous pathogens) of the hosts, did not analyze the enduring effects of trained immunity, and/or did not support their observations by detailed explanation of the underlying biochemical and molecular mechanism(s) involved. Studies systematically examining all these parameters in a comprehensive manner are crucial to gain a more holistic and realistic understanding of the existence of trained immunity in invertebrates.

There is accumulating evidence that epigenetic regulatory mechanisms are a central element in the function of the immune system, allowing an appropriate gene expression pattern in immune cells in response to internal or external environmental cues, including pathogenic factors^10^. Some of these mechanisms have been elucidated and include DNA methylation^11^, histone modifications and chromatin-remodeling proteins^12^, and DNA silencing by noncoding RNAs^13^. Epigenetic modifications have been shown to support information storage during innate immune memory in different experimental model systems^14^. For instance, in plant models, which do not possess adaptive defense system, epigenetic programming at the level of histone protein modifications has been proposed as the molecular mechanism responsible for long-term memory for innate immunity^15^,^16^. These findings triggered the hypothesis that epigenetic programming of the immune-related genes might be the mechanistic basis for mediating the phenomenon of trained immunity in invertebrates.

In this study, using the well-developed host-pathogen model system, the brine shrimp *Artemia franciscana* (an aquatic invertebrate) and its pathogenic bacterium *Vibrio*^17^, we aimed to unambiguously proof the likelihood of trained immunity, examining the presence or absence of enduring memory against homologous and heterologous antigens (*Vibrio* spp.) during a transgenerational study. Furthermore, we also aimed to unravel the underlying mechanisms for the possible existence of trained immunity in invertebrates by focusing on the immunological pathways and epigenetic mechanisms.

## Results

### Progeny of *V. campbellii*-exposed ancestors exhibit increased resistance against challenge with homologous bacterial strain

**Figure 1:**
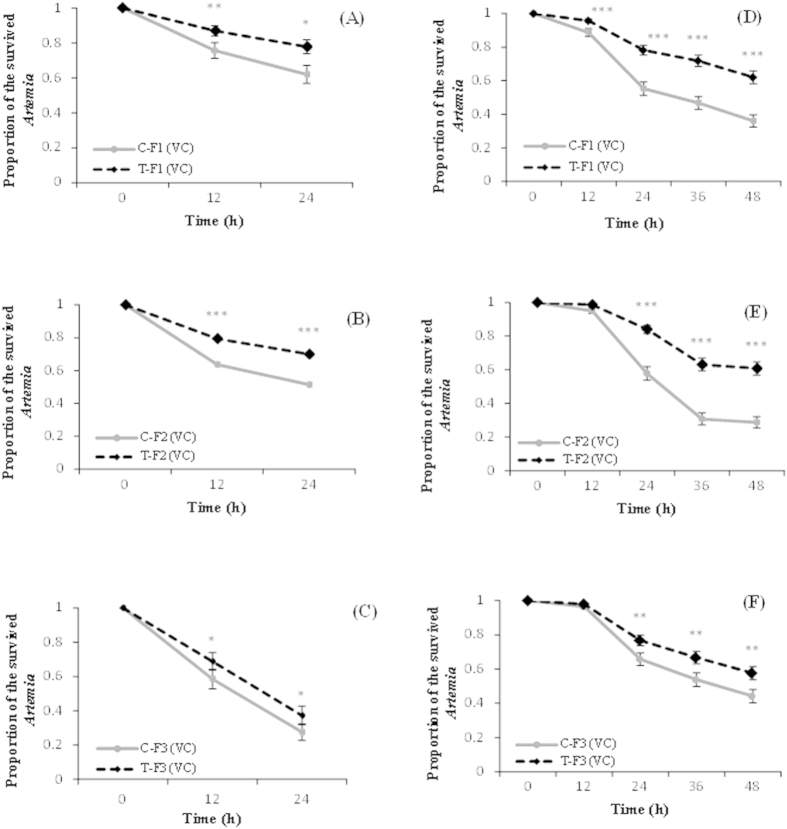
Progeny of *V. campbellii*-exposed ancestors exhibit increased resistance against challenge with homologous bacterial strain. See Fig. 9 for explanation of the treatment (T) and control (C) groups. (**A–C**) At every generation [F1 (**A**), F2 (**B**), and F3 (**C**)], the ovoviviparously produced larvae (at instar II stage) were challenged with *V. campbellii* at 10^7 ^cells/mL and survival was scored at 12 h intervals. (**D–F**) Cysts from generations F1 (**D**), F2 (**E**), and F3 (**F**), were hatched simultaneously under axenic conditions, after which the larvae were challenged with *V. campbelii*, and survival was scored (common garden test). Values are presented as means ± SE (n = 3). Asterisks represent significant difference between the control and treatment *(P < 0.05), **(P < 0.01), ***(P < 0.001).

To determine the occurrence of adaptivity in the defense system of the invertebrate *Artemia*, we first analyzed the phenotype of induced resistance in the F1 progeny, produced ovoviviparously, towards pathogenic *V. campbellii* following challenge of the parental F0 population with exposure to the same pathogenic bacterial strain. As shown in Fig. 1A, the unexposed T-F1 progeny derived from T-F0 *Artemia* exposed repeatedly at early stages to *V. campbellii,* exhibited increased resistance towards *V. campbellii* challenge, as was evidenced by significantly higher survival than the respective progeny of control C-F1 *Artemia*. To examine the durability of the transgenerational resistance, the F1 progeny from both control and treatment groups were grown till adult under stress-free (no *Vibrio*-based challenge) conditions, and bred to produce F2 and F3 generations. The results showed that the higher survival phenotype persisted even in the T-F2 and T-F3 progenies, whose grandparents and great grandparents, respectively, were exposed at early stages to *V. campbellii* (Fig. 1B,C).

In the following experiment, we validated the findings obtained in the above survival test by carrying out a common garden experiment under gnotobiotic conditions. It is because in the above test, the microbial communities (in terms of activity and composition) that remain associated with the host, and the size and age of the progenies across the generations might not be the same. The observed responses could therefore be an artefact of these varying factors rather than the genuine effect of the treatment conditions. In this common garden experiment, *Artemia* cysts from all 3 (F1 to F3) generations were hatched simultaneously in an axenic way^18^, and the obtained axenic larvae were challenged with *V. campbellii* as described above. The results showed a similar survival trend as observed for the animals from ovoviviparous reproduction (Fig. 1D–F). These results suggest that the defense system of the invertebrate *Artemia* can adapt, providing enhanced and enduring protective effects against subsequent *Vibrio* infection.

### Progeny of *V. campbellii*-exposed ancestors exhibit no increased resistance against challenge with heterologous *Vibrio* strain

**Figure 2:**
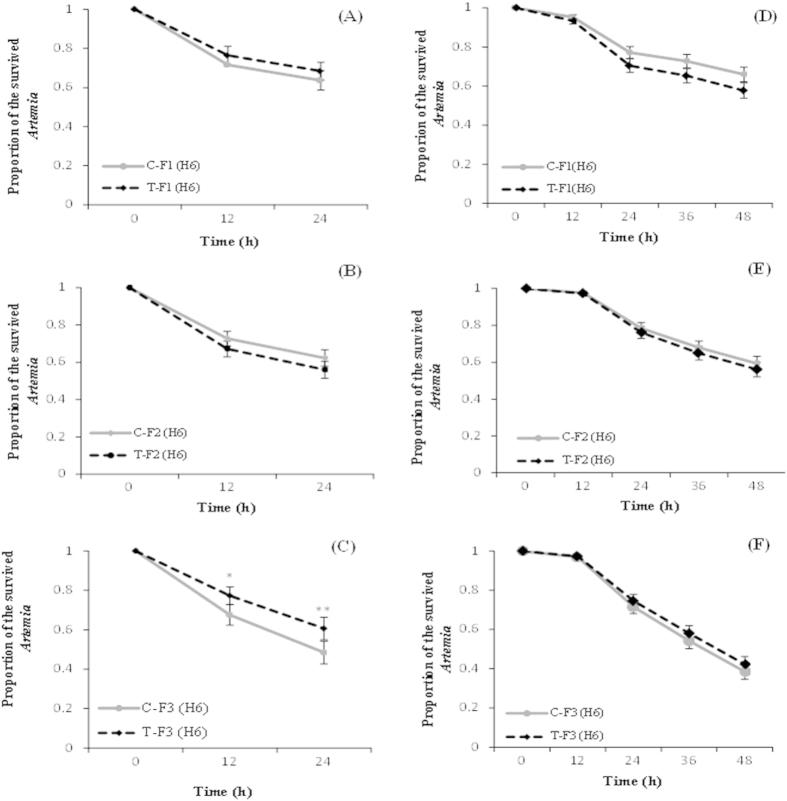
Progeny of *V. campbellii*-exposed ancestors exhibit no increased resistance against challenge with heterologous *Vibrio* strain. See Fig. 9 for explanation of the treatment (T) and control (C) groups. (**A–C**) At every generation [F1 (**A**), F2 (**B**), and F3 (**C**)], the ovoviviparously produced larvae (at instar II stage) were challenged with H6 at 10^7 ^cells/mL and survival was scored at 12 h intervals. (**D–F**) Cysts from generations F1 (**D**), F2 (**E**), and F3 (**F**), were hatched simultaneously under axenic conditions, after which the larvae were challenged with H6, and survival was scored (common garden test). Values are presented as means ± SE (n = 3). Asterisks represent significant difference between the control and treatment *(P < 0.05), **(P < 0.01), ***(P < 0.001).

Next, we aimed to determine whether progeny of *V. campbellii*-exposed ancestors could build resistance against challenge with heterologous strain of *Vibrio*. To this end, we carried out another *Vibrio* challenge test. Here the F1 to F3 progenies produced ovoviviparously from F0 *Artemia* that were exposed to *V. campbellii* at early stage were challenged with another pathogenic *Vibrio* strain H6. We found that the unexposed T-F1 progeny derived from T-F0 *Artemia* exposed to *V. campbellii* did not exhibit any significant improvement in their survival upon challenge with H6 strain compared to the same-generation progeny of control parents (C-F1 progeny) (Fig. 2A). Similar result was observed at the F2 generation (Fig. 2B). However, at F3 generation, the survival of the T-F3 progeny whose great grandparents were exposed to *V. campbellii* was significantly higher than that of the C-F3 progeny of control parents (Fig. 2C).

We confirmed the results of the above survival test by conducting the experiment under common garden and gnotobiotic conditions. We found that none of the progenies from the T-F1 to T-F3 generations of (F0) *Artemia* that were exposed to *V. campbellii* exhibited increased resistance towards the pathogenic H6 strain, as was evidenced by the absence of a significant difference in the survival compared with the respective progenies of the control parents (Fig. 2D–F). These data from both the common garden experiment and the generation-specific challenge tests, together with the data from the *Vibrio*-resistance test reported above suggest that *Artemia* defense system shows small degree of discrimination between the two *Vibrio* strains.

### Progeny of *V. campbellii*-exposed ancestors exhibit alteration in their reproductive behavior

**Figure 3:**
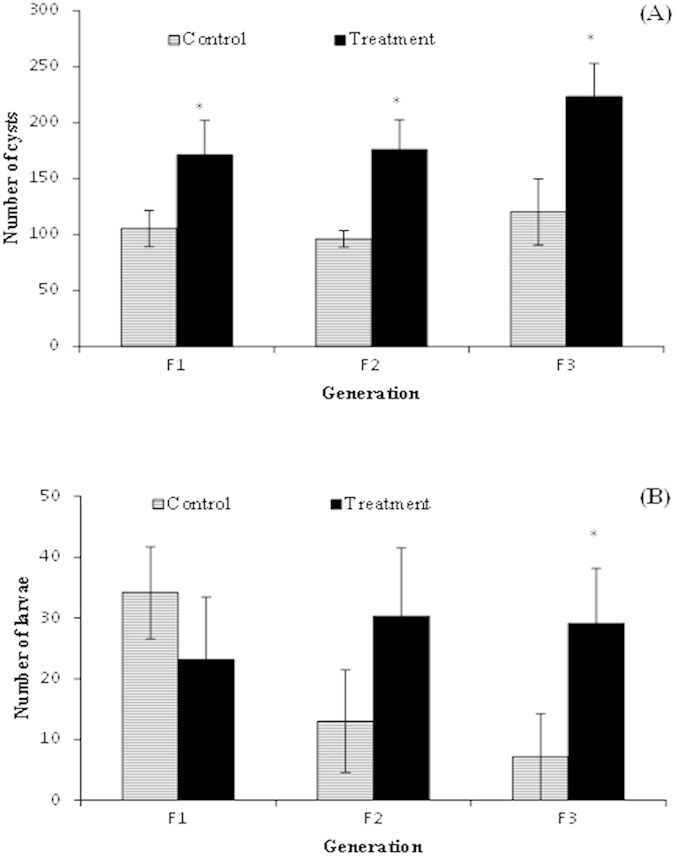
Progeny of *V. campbellii*-exposed ancestors exhibit alteration in their reproductive behavior. See Fig. 9 for explanation of the experimental groups. Adult *Artemia* couples from F1 to F3 generation of the control and treatment groups reared under common garden conditions experiment were distributed, in 8 replicates, in Falcon tubes that contained seawater of 80 g/L salinity. The production of (**A**) cysts and (**B**) larvae from each couple were monitored twice a week for a period of 3 weeks after their first reproduction. Values are presented as means ± SE (n = 8). Asterisks represent significant difference between the control and treatment *(P < 0.05).

Previous study reported that the occurrence of trained innate immune responses in invertebrates is costly and can cause marked effects on numerous life-history traits (e.g. fecundity, egg size) of the animal^6^. We next investigated whether *Vibrio* exposure of parental *Artemia* (T-F0) bears any costs of the progeny for 3 successive generations. For that, we measured the reproductive behavior in terms of larval production and fecundity of the progeny from the F1 to F3 generations of *Artemia* that were challenged with *Vibrio*. The results showed that the T-F1 to T-F3 female progenies, whose ancestors were challenged with *Vibrio*, produced significantly higher number of cysts (dormant eggs) than their respective progeny from the control ancestors (Fig. 3A). For the number of larvae produced ovoviviparously by the progenies from F1 to F3 generation, we did not observe any significant difference between the control and treatment progenies at the F1 and F2 generation. However, interestingly this response appeared to reverse in the F3 generation, where the T-F3 progeny exhibited a significant 4-fold increase in the number of larval production compared to the C-F3 progeny of the control parental group (Fig. 3B). These results indicate that in response to *Vibrio* exposure, *Artemia* may adaptively adjust the reproductive phenotype of their progenies for 3 successive generations.

### *Vibrio campbellii*-exposed parental generation and their unexposed progenies showed increased levels of *hsp70* and *hmgb1* transcripts

**Figure 4:**
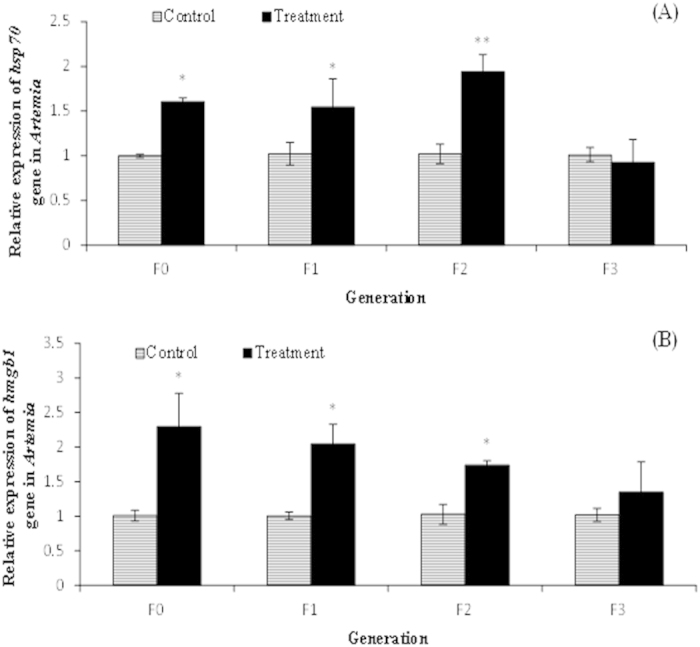
*Vibrio campbellii*-exposed parental generation and their unexposed progenies showed increased expression of *hsp70* and *hmgb1* genes. See Fig. 9 for explanation of the experimental groups. Total RNA extracted from F0 to F3 juveniles, reared under common garden conditions, was analyzed for expression of (**A**) *hsp70* and (**B**) *hmgb1* genes by qPCR assay. At each generation, the expression of *hsp70* or hmgb1 gene in the control group was set at 1.0 and all other data points were normalized accordingly using the equation of Pfaffl *et al.* (2002)^52^. The actin gene was used as an internal control. Error bars represent the standard errors of three biological replicates. Significant differences between the treatment and control at respective generation are indicated by *(P < 0.05), **(P < 0.01).

Because the signaling molecules heat shock protein 70 (HSP70) and high mobility group box 1 (HMGB1) are associated with the induction of resistance within the host *Artemia* against bacterial infection^19^, we next sought to investigate whether these signal molecules are associated with the observed acquired memory-like resistance against pathogenic *V. campbellii*. We addressed this by analyzing the expression levels of *hsp70* and *hmgb1* genes by employing RT-qPCR. As shown in Fig. 4, the expression levels of *hsp70* and *hmgb1* in the *Vibrio*-exposed parental *Artemia* (T-F0) were significantly higher by respectively 1.6-fold and 2.3-fold (P < 0.05) relative to the unexposed C-F0 parental group. Increased expression levels of *hsp70* and *hmgb1* were also detected in the T-F1 (by 1.5- and 2-fold, respectively, P < 0.05) and T-F2 (by 1.9- and 1.7-fold, respectively, P < 0.05) progenies, whose ancestors were challenged with *Vibrio*. However, in the F3 generation, no significant difference was found in the expression levels of *hsp70* and *hmgb1* between the C-F3 control and T-F3 treatment groups. These results might indicate that HSP70 and HMGB1 signaling molecules are associated, at least in part, with orchestrating the induction of acquired memory-like resistance against *V. campbellii* as observed in the survival tests.

### *Vibrio campbellii*-exposed parental generation and their unexposed progenies did not exhibit increased levels of *dscam* and *lgbp* transcripts

**Figure 5:**
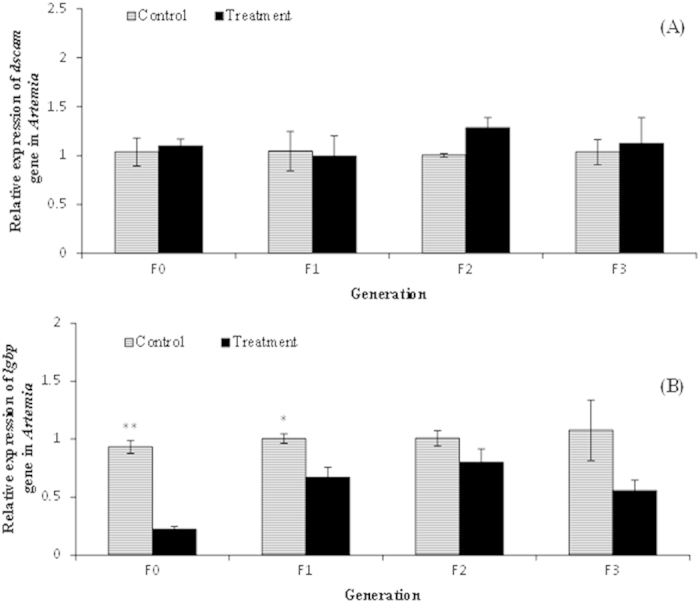
*Vibrio campbellii*-exposed parental generation and their unexposed progenies did not exhibit increased expression of (**A**) *dscam* and (**B**) *lgbp* genes. See Fig. 4 for explanation of the experimental groups and methodology. Error bars represent the standard errors of three biological replicates. Significant differences between the treatment and control at respective generation are indicated by *(P < 0.05), **(P < 0.01).

Previous studies reported that pattern recognition receptors (PRR) are involved in the first step of immune responses in invertebrates by binding to highly conserved pathogen structures, such as peptidoglycans or lipopolysaccharide (LPS) from bacteria, or to danger associated molecular patterns, such as HSPs and HMGB1^20^,^21^. Since the expression levels of *hsp70* and *hmgb1* were increased in the treated parental generation as well as in their successive offspring, we next explored the involvement of PRRs in the induction of resistance phenotypes in the T-F1 to T-F3 progenies of challenged F0 *Artemia*. To address this, we focused on the transcripts of two important PRRs in invertebrates i.e. down syndrome cell adhesion molecule (*dscam*) and lipopolysaccharide- and beta-1,3-glucan-binding protein (*lgbp*) that have been shown to be responsive to bacterial infection and are involved in various ways in the biological defense mechanisms in invertebrates^22^. Surprisingly, we observed no significant increase in the transcript levels of *dscam*, neither in the *Vibrio*-exposed T-F0 parental generation nor in their successive generation T-F1 to T-F3 progenies (Fig. 5A). The transcript levels of *lgbp* also did not increase significantly in any of the treatment (T-F0 to T-F3) groups compared to their respective controls (Fig. 5B). In contrast, the transcript levels in the T-F0 *Artemia* were downregulated by 4.2-fold relative to the C-F0 group (P < 0.05). The T-F1 progeny of parents that were exposed to *Vibrio* also exhibited significantly lower expression levels for this gene relative to the respective T-F1 progeny of the control parental group (1.5-fold, P < 0.05). In the T-F2 and T-F3 progenies, a trend towards a decrease in the transcript levels of *lgbp* relative to their respective controls was observed, but the difference was not statistically significant (P > 0.05). These observations suggest that the immune receptors *dscam* and *lgbp* are not associated with the acquired memory-like resistance phenomenon in the T-F1 to T-F3 progenies of *V. campbellii-*exposed F0 *Artemia.*

### *Vibrio campbellii*-exposed parental generation and their unexposed progenies exhibited changes in the transcription of innate immune-related genes

**Figure 6:**
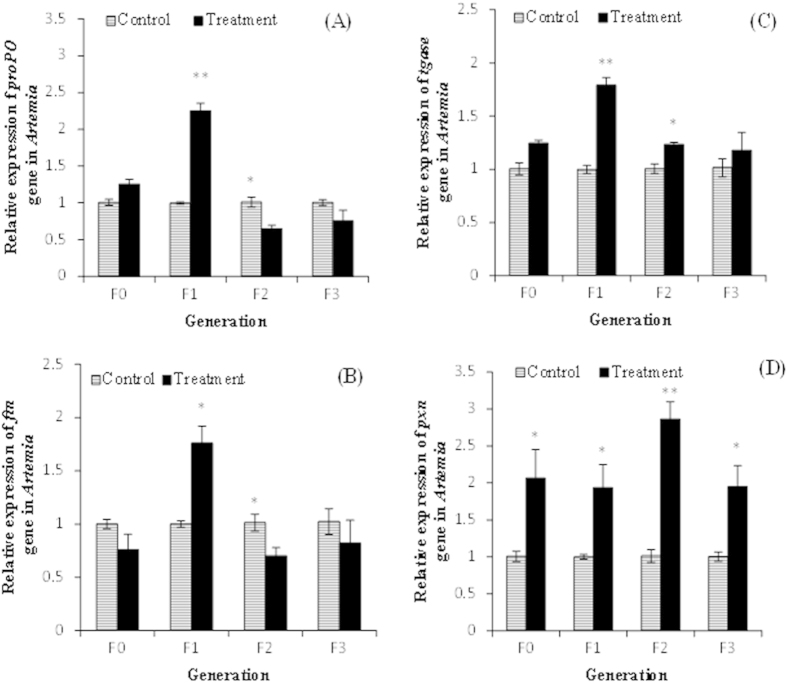
*Vibrio campbellii*-exposed parental generation and their unexposed progenies exhibited changes in the expression levels of (**A**) *proPO*, (**B**) *ftn*, (**C**) *tgase* and (**D**) *pxn* genes. See Fig. 4 for explanation of the experimental groups and methodology. Error bars represent the standard errors of three biological replicates. Significant differences between the treatment and control at respective generation are indicated by *(P < 0.05), **(P < 0.01).

Next we investigated the contribution of humoral immune responses to the induction of acquired resistance traits in the T-F1 to T-F3 progenies of the F0 *Artemia*. To this end, we analyzed the expression of a set of four innate immunity-related genes i.e. prophenoloxidase (*proPO*), ferritin (*ftn*), transglutaminase (*tgase*) and peroxinectin (*pxn*) that were previously reported to be involved in inducing resistance in invertebrate (including *Artemia*) against bacterial infection^23^,^24^. As shown in Fig. 6A, there was no significant increase in the expression level of the *proPO* gene in the *Vibrio*-exposed parental T-F0 *Artemia* relative to the unexposed C-F0 parental group. In the F1 generation, progeny of the parents that received *V. campbellii* challenge exhibited a 2.3-fold increase in the expression level of *proPO* gene relative to the corresponding C-F1 control (P < 0.01). The *proPO* expression level in the T-F2 progenies down regulated significantly (P < 0.05) by 1.5-fold relative to the control C-F2 progeny. However, in the F3 generation, the expression level of *proPO* in the T-F3 progeny did not differ significantly from that in the corresponding C-F3 control progeny.

Similar to what was observed for the *proPO* gene, the expression level of *ftn* in the T-F0 parental *Artemia* remained at the same level as in the C-F0 parental group (Fig. 6B). In the F1 generation, the T-F1 progeny exhibited a significant (P < 0.05) 1.8-fold increase in the *ftn* expression level relative to the control C-FI progeny. In the F2 generation, the expression level in the T-F2 progeny declined significantly by a factor of 1.4-fold. However, in the F3 generation, the expression level in the T-F3 progeny was the same as in the respective C-F3 control.

The *tgase* gene expression level in the T-F0 progeny remained unaltered in comparison to the respective C-F0 control progeny (Fig. 6C). However, in the F1 and F2 generations, the expression levels in the T-F1 and T-F2 progenies increased significantly by respectively 1.8-fold (P < 0.01) and 1.2-fold (P < 0.051) relative to the respective C-F1 and C-F2 controls. In the F3 generation, no significant difference in the *tgase* expression level was observed between the C-F3 control and T-F3 treatment groups.

The *pxn* expression level in the *V. campbellii* exposed parental *Artemia* (T-F0) was significantly higher (2-fold, P < 0.05) than in the unexposed C-F0 parental group (Fig. 6D). A significantly increased expression of *pxn* was also detected in the T-F1 to T-F3 progeny, whose ancestors were exposed to *V. campbellii*. The increase was by respectively 1.9-fold (P < 0.05), 2.7-fold (P < 0.01) and 2-fold (P < 0.05) at the F1, F2 and F3 generations relative to the respective controls. Taken together, these results suggest that in response to immune challenge of the parental *Artemia* with *V. campbellii* exposure, the *proPO, tgase* and *ftn* and *pxn* immune genes were induced in a differentially manner both within the parental generational as well as in the 3 successive T-F0 to T-F3 generations.

### *Vibrio campbellii*-exposed parental generation and their unexposed progenies exhibited a stochastic pattern in the level of histones H3 and H4 acetylation

**Figure 7:**
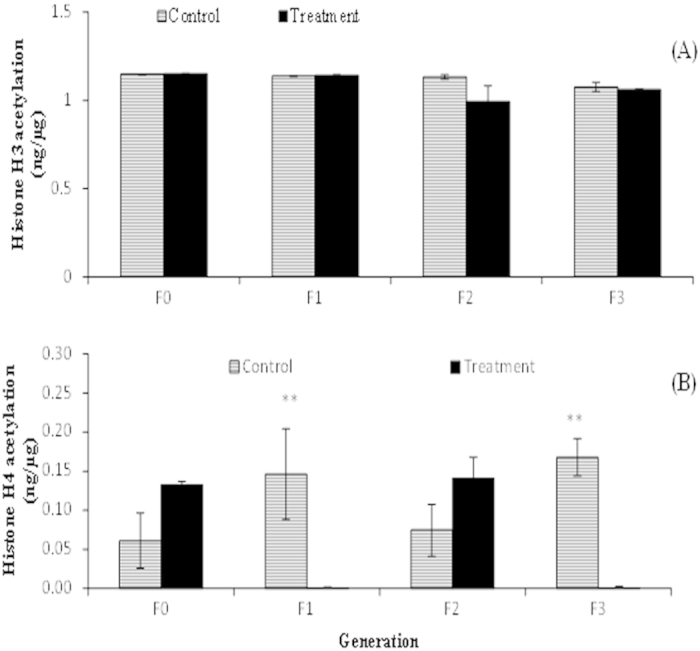
*Vibrio campbellii*-exposed parental generation and their unexposed progenies exhibited stochastic pattern in the acetylation level of histones H3 and H4. See Fig. 9 for explanation of the experimental groups. Total histone (3 μg) extracted from F0 to F3 juveniles, reared under common garden conditions, was analyzed for total histone H3 (**A**) and H4 (**B**) acetylation using a flurometric kit. Values are presented as means ± SE (n = 4). Asterisks represent significant difference between the control and treatment at respective generation **(P < 0.01).

Since acetylation of histone proteins is critical involved in the transcriptional (up)regulation of defense-related genes in organisms^25^, we next aimed to unravel whether modification of histone H3 and H4 proteins is the underlying mechanism behind the phenomenon of acquired memory-like resistance phenotypes in *Artemia* as observed during our transgenerational study. As shown in Fig. 7A, we observed no significant increase in the acetylation levels of histone H3 protein, neither in the *Vibrio*-exposed T-F0 parental generation nor in the successive generation, unexposed T-F1 to T-F3 progenies. In regard to histone H4, a stochastic pattern of acetylation level was observed across T-F0 to T-F3 generations of *Artemia* (Fig. 7B). This means a relatively higher level of acetylated H4 in the T-F0 parental *Artemia* than in the C-F0 parental group (2.2-fold; P > 0.05), significantly lower level in the T-F1 progeny compared to the C-F1 control progeny (15-fold; P < 0.05), again considerably higher in the T-F2 progeny compared to C-F2 control progeny (1.9-fold; P > 0.05), and then significantly lower level in the T-F3 progeny compared to the corresponding control progeny (17-fold; P > 0.05).

### *Vibrio campbellii*-exposed parental generation and their unexposed progeny exhibited a stochastic pattern in the trimethylation levels of histone H3 at lysine 4 tail (H3K4me3)

**Figure 8:**
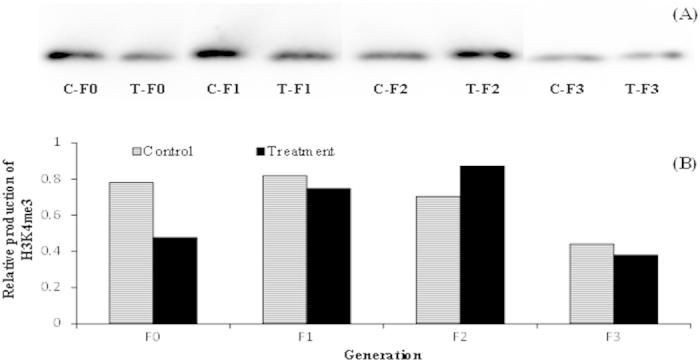
*Vibrio campbellii*-exposed parental generation and their unexposed progenies exhibited stochastic pattern in the trimethylation levels of histone H3 at lysine 4 tail (H3K4me3). See Fig. 9 for explanation of the experimental groups. (**A**) Total histone extracted from F0 to F3 juveniles, reared under common garden conditions, was resolved in SDS-PAGE gel, transferred to polyvinylidene fluoride membrane and then probed with anti-H3K4meE primary antibody. 15 μg of *Artemia* histone protein was loaded in each lane. HeLa cells (6 μg) were loaded onto one well to serve as a positive control and for calculating the amount of histone H3K4me3 in the sample. Cropped blot image was shown and the full-length blot was presented in Supplementary Figure S1. (**B**) Quantitative analysis of histone H3K4me3 levels in *Artemia* is presented relative to H3K4me3 trimethylation level in HeLa cells.

To gain more insights, we next attempted to analyze histone H3K4me3 which is associated with giving rise to detectable transcription of genes^26^. As shown in Fig. 8, the induction level of H3K4me3 was markedly lower in the T-F0 parental *Artemia* than that in the control C-F0 parental group. Relatively lower level of H3K4me3 was also detected in the T-F1 progeny compared to the C-F1control. However, at F2 generation the H3K4me3 level increased considerably in the T-F2 progeny, whose grandparents were exposed to *V. campbellii*, compared to the corresponding C-F2 control group. The induction level of H3K4me3 in the T-F3 progenies appeared to decrease relative to that in the C-F3 progenies.

## Discussion

In this study, we addressed the possible occurrence of trained immunity in invertebrates and the underlying molecular mechanisms involved in a more comprehensive approach by carrying out a transgenerational study, wherein a population of parental *Artemia* was challenged at early stages of their life with *V. campbellii*, and the resistance of 3 successive, unexposed generation progenies towards the same or different bacterial strain was analyzed. Our results provided clear evidence suggesting the occurrence of memory in the innate immune system of the invertebrate *Artemia*, as manifested by increased resistance of the T-F1 to T-F3 progenies to *V. campbelli* provided their ancestors were exposed to the same strain of *Vibrio*. The bacterial-resistant tests across generations were conducted under both gnotobiotic (germ-free) and conventional (germ-associated) conditions excluding the possibility of confounding factors, such as differential microbial community e.g. across generations. Under both these experimental conditions, we observed similar phenotypes of acquired-resistance in *Artemia*. Our results also indicated that innate immune system of *Artemia* could discriminate (in terms of building resistance) to some extent between the two *Vibrio* strains. The observed effect could possibly be due to differences in the composition of the conserved microbial molecules (i.e. PAMPs) present on these *Vibrio* strains that differentially trained the innate immune machineries of *Artemia*. It is however important to mention that the structural difference between the two *Vibrio* strains on a molecular level is currently unknown. Efforts are currently underway in our laboratory to identify the differences between the two strains by using MALDI-TOF MS analysis in an attempt to address these possibilities. Nevertheless, to the best of our knowledge, this is the first report to verify under both germ-free and germ-associated conditions in a transgenerational study the occurrence of adaptive-like features in the innate immune system of invertebrates. It is interesting to mention here that the effects seen across the generations in our study is less likely due to selection on the parental population. It is because, in an another study, which is an extension of the present study, we observed that upon *V. campbellii*-based immune challenge of an apomictic parthenogenetic *Artemia* population produced from a single female (i.e. a female parthenogenetic *Artemia* population clone, which has no other mechanism for genotypic change^17^), its 3 successive, naïve generation progenies exhibit similar increased resistance towards the same bacterial challenge^17^. However, it is also not unlikely that the observed phenotypic responses across the generations is due to genetic mutational event (such as bacteria-facilitated single-nucleotide polymorphisms) but this remains unknown and needs further investigation.

As is true for the adaptive immune response in general, generation of transgenerational immune defense in invertebrates is costly^27^ and can result in trade-offs with other life-history traits, such as reproduction and development^28^. For instance, in the invertebrate red flour beetle, it was found that trained immunity of the parental generation markedly reduced reproductive fitness (i.e. low fecundity) of the offspring^6^. However, in our study, the results showed that there is no cost of trained immunity of the parental generation for the net reproductive success/fitness of the progeny for 3 successive generations, suggesting that the observed trained immunity in *Artemia* occurred without trade-off of other fitness traits.

The observed occurrence of trained immunity in *Artemia* could be a result of various events. Our results suggested that the observed phenomenon could be associated, at least in part, with the induction of the signaling molecules HSP70 and HMGB1 since a positive correlation between elevated *hsp70* and *hmgb1* transcript levels and increased resistance phenotypes was observed in the T-F1 to T-F3 generation progenies. This suggestion is consistent with the known important roles that HSP70 and HMGB1 proteins (that get induced during pathogenic stress response) play in defining the resistance of organisms against stressor^19^ by performing multifaceted functions, such as acting as molecular chaperone for protein and DNA, respectively, functioning as danger associated molecular pattern (DAMP) during inflammation and various cellular processes^29^,^30^, and/or participating in the activation of cell surface innate immune receptors, thereby modulating many aspects of host’s immune responses^20^,^21^. The reason that unexposed T-F3 progeny whose ancestors were exposed to *V. campbellii* had increased resistance towards subsequent *V. campbellii* challenge despite an absence in increased level of *hsp70* and *hmgb1* gene expression is less clear, although one could argue the expression/production of other molecular chaperones, such as HSP40, HSP60 and HSP90 in this progeny may provide a partial explanation^18^,^31^.

Previous studies which determined the role of HSP70 and HMBG1 proteins in mediating immune responses in other model systems reported that both HSP70 and HMBG1 initiate inflammatory responses in many disease conditions via a receptor-mediated mechanism^20^,^32^. The pathogen recognition receptor DSCAM is a highly plastic one that has previously been shown to mount adaptive-like immunity in invertebrates through specific splicing during the pathogen encounters^33^,^34^. For instance, in a study on the invertebrate *Anopheles gambiae*, it was shown that the DSCAM genome that has a complex organization with 101 exons produces over 31,000 potential alternative splice forms with different combinations of adhesive domains and interaction specificities, and reacts specifically to pathogen thereby protecting the animals against infection^35^. LGBP is another PRR which was shown to play crucial roles in generating innate immune defense against Gram-negative bacteria (like *V. campbellii*) in aquatic invertebrates^36^. In our study, we did not see a clear link between expression of the genes encoding for the *hsp70* and *hmbg1* signaling molecules and for the *dscam* and *lgbp* PRRs. These results suggest that *hsp70* and *hmbg1* may mediate their effects on the generation of trained immunity in *Artemia* through other immunological receptors rather than through the tested DSCAM and LGBP^37^.

Despite the absence of significant changes in the expression levels of *dscam* and *lgbp*, a few downstream genes known to be involved in the humoral innate immune response showed altered expression levels across three successive generations in response to *Vibrio* challenge of their ancestors (see Fig. 6). Among the immunity-related genes, those encoding for the immune effectors proPO, Tgase and ferritin were upregulated in a stochastic manner across the T-F0 to T-F3 generations. However, the gene encoding for peroxinectin remained elevated both in the parental generation in response to *Vibrio* exposure as well as in their successive generation, unexposed progenies. The *proPO* and *tgase* genes are important constituents of the innate immune repertoire in invertebrates that confer protection against invading pathogens by their role in melanization and coagulation, respectively^18^,^38^. The *ftn* gene encoding the protein ferritin participates in invertebrate’s innate humoral response to bacterial infection by mechanism of withholding iron, an essential nutrient for growth and survival of bacteria^39^. The peroxinectin-encoding gene *pxn* on the other hand is multifunctional immune component in invertebrates involved in biological processes, such as cell adhesion, hemocyte degranulation, opsonization, peroxidase activity and transduction pathway regulating the expression of antimicrobial peptides^40^,^41^. From our results reported above, we can suggest that there is a non-linear relationship between the immunity-related genes (except for *pxn*) and the observed acquired resistance traits in the T-F1 to T-F3 generations. In invertebrates, each of the effector systems involved in the immune response may carry a different cost when activated and their relative expression may shape the cost of the whole immune response to a standard challenge. The observed variation in the degrees of expression between the immune effectors might be an adaptive defensive strategy of the progenies for subsequent pathogenic challenge, while minimizing the potential costs of the immune response^42^, as observed in our study (see Fig. 3).

For offspring to profit from a past experience from the parents, the latter have to be able to perceive the specific stress, they have to store this information, retain and transmit it to the progenies. To benefit from this information the progenies have to be able to retrieve the information and translate it into appropriate reactions. While the mechanistic insights into the molecular processes behind the occurrence of trained immunity are not well understood in invertebrates, a few studies have pointed out to the possible involvement of epigenetic mechanisms in trained immunity^43^,^44^,^45^. Histone modifications (in terms of acetylation or methylation) are examples of epigenetic mechanisms; indeed, such modifications in the promoters of defense genes have been shown to correlate with transgenerational induced resistance against abiotic^46^ and biotic stresses^47^ in the *Arabidopsis* plant models (organisms that are devoid of adaptive immunity similar to invertebrates). In our earlier study, we have demonstrated that, on exposure to an abiotic stressor, a parental population of parthenogenetic *Artemia* experiences an increase resistance against pathogenic *V*. *campbellii* and this acquired resistance phenotype were transmitted to three successive generations, none of them were exposed to the parental stressor^31^. Results also suggested that the transgenerational inheritance of the increased resistance traits was mediated by increased acetylation states of the epigenetic marks histones H3 and H4^31^. In the present study, we observed alterations in the acetylation and methylation levels of H4 and H3K4me3 histones, respectively, but not of histone H3, across the T-F0 to T-F3 generations; however, these epigenetic changes did not correlate with the transcription levels of immune genes analyzed as well as with the observed acquired resistance traits. As mentioned above, we have analyzed total histone acetylation/methylation levels and not histone modifications on specific immune-genes (e.g., *proPO, tgase, pxn* and *ftn*). This result, however, does not eliminate the possibility that the observed adaptive-like features in the defense system of *Artemia* are due to epigenetic modifications at specific immune gene(s). It is also important to mention that besides functioning as signaling molecules, HMGB1 is a non-histone chromatin-associated protein, which function is to stabilize nucleosome (histone/DNA complex) formation and to act as transcription-factor like protein that regulates gene expressions by bending DNA and promoting access to transcriptional proteins on specific DNA targets^48^. The association of this non-histone mark in the appropriate regulation of the immune genes, leading to the induction of resistance at each generation cannot be excluded. Currently, little is known on how these histone and non-histone proteins, together with DNA methylation (another epigenetics mark responsible for mediating immune priming^49^) interact among each other in regulating appropriately the expression of genes in the immune cells that leads to trained immunity in invertebrates. These mechanistic details need further verification.

In conclusion, we provide evidence that the innate defense system of the invertebrate *Artemia* has adaptive features and that it has the capacity to induce long lasting protective effects against subsequent challenge with the same pathogen. This process of trained immunity likely represents a paradigm shift in immunity, as it demonstrates the existence of immunological memory in the absence of adaptive immune responses. Better insights into the role of epigenetic reprogramming of the immune system in invertebrates may have important consequences for the design of drugs for invertebrates of commercial importance (such as shrimps) to combat diseases challenging these high-food value organisms.

## Methods

### The experimental animal axenic *Artemia*

The brine shrimp *Artemia franciscana* cysts (dormant embryos) originating from the Great Salt Lake, Utah, USA (INVE Aquaculture, Belgium) were used to produce the parental generation. To avoid any possible microbial contamination from the previous environment, *Artemia* cysts were hatched under axenic (germ-free) conditions as described previously^23^. The sterile decapsulated embryos were transferred to 2-L glass bottles containing 35 g/L of sterile artificial seawater. Following incubation at 28 °C under constant illumination for 48 h (or day 2 posthatching), the emerged instar II larvae (developmental stage at which the mouth is open for ingestion of food) were used for the experiments.

### Bacterial strains and growth conditions

Two pathogenic bacterial strains, *Vibrio campbellii* strain LMG21363 and *Vibrio* strain H6 (belonging to the Harveyi clade of *Vibrio*) were used in the *Artemia* challenge study. Both these strains are Gram-negative bacteria that are ubiquitous in the marine environment. The *V. campbellii* strain, originated from the stock cultures of *Artemia* Reference Center, Ghent University, was known to cause life threatening vibriosis (bacterial disease) in wild and cultured aquatic animals, including *Artemia*^50^. Strain H6 was obtained from a hatchery in Rio Grande do Norte (Natal-Area, Brazil) and was originally isolated from pacific white shrimp *Penaeus vannamei*. H6 was chosen for reason of its higher virulence, than *V. campbellii* strain LMG21363, towards *Artemia*^51^. Both *Vibrio* strains were stored in 40% glycerol at −80 °C until use. For challenge experiments, the strains were cultured under optimum conditions as previously described^31^,^51^ and immediately used. Bacteria cell numbers were quantified spectrophotometrically as described previously^18^.

### Challenge of parental (F0) generation

**Figure 9:**
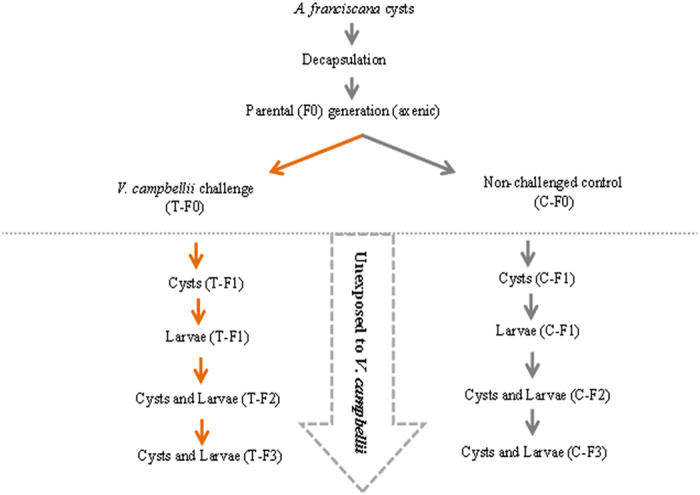
Scheme of the experiment. *Artemia* cysts were hatched under axenic conditions to produce the parental (F0) generation larvae. The F0 larvae were divided into 2 groups. One group was exposed to *V. campbellii* ahead of the reproductive period (T-F0) as described in the methodology. The other group was grown unexposed, under normal culture conditions at 28 °C (C-F0). Approximately 26 days posthatching, the adult parental (F0) females from the treatment (T-F0) and control (C-F0) groups produced their next generation cysts i.e., T-F1 and C-F1, respectively. The cysts were hatched to produce their corresponding larvae i.e., T-F1 and C-F1. The F1 larvae from both groups were further cultured isothermally at 28 °C, without being given *V. campbellii* exposure, to maturity, after which the F2 larvae were collected. The experiment was continued until, and including, the F3 generation.

A total of 8000 instar II larvae (day 2 post hatching) were equally divided into two groups (treatment and control). For each group, 3 replicates were maintained. The scheme of the experiment is described in the supplementary information (see Fig. 9). Each group was maintained in 2-L glass bottles containing sterile artificial seawater (35 g/L), maintained at 28 °C under constant illumination (approximately 27 μmoL/m^2^/s) and aeration. The treatment group (on day 2 posthatching) was exposed to pathogenic *V. campbellii* strain at a concentration of 10^7 ^cells/mL for a period of 4 days. On day 6 posthatching, the viable larvae from the bottles were collected over a sieve (250 μm), rinsed several times with sterile seawater to wash away the bacteria associated with the larvae, re-suspended in new sterile 2-L glass bottles that contained sterile artificial sea water, and then allowed to recover for 3 days. On day 10 posthatching, the larvae were washed in a similar way as described above and then exposed to the same concentration of *V. campbellii* for another 4 days. The control group, unexposed to *V. campbellii*, went through the same handling process. The *V. campbellii* exposure to *Artemia* larvae was continued till day 14 posthacthing ahead of the reproductive period (under standard laboratory conditions, *Artemia’s* uterus develop on day 16 posthatching). Only early life stages of *Artemia* were exposed to *V. campbellii* to ensure that the uterus carrying the cysts/embryos was not directly exposed to the pathogenic stress conditions. On day 17 posthatching, the salinity of the rearing water was increased from 35 g/L to 70 g/L in order to inhibit the growth of *V. campbellii*. Under non-optimal environmental conditions, such as high salinity or low oxygen, *Artemia* switch from an ovoviviparous to an oviparous mode of reproduction^31^. On day 20 posthatching, therefore, the salinity was further increased to 80 g/L to instigate cysts production by oviparous reproduction^31^. Throughout the culture period, the animals were fed *ad libitum* everyday with live green microalgae *Tetraselmis suecica* (Dunstaffnage Marine Laboratory, Oban, UK).

### Production and collection of the progenies as larvae and cysts

Approximately 26 days posthatching, adult females (F0 generation) in both control and treatment groups produced larvae and cysts (F1 generation). As the F1 larvae were produced in the parental generation environmental conditions, they were therefore discarded, and only the F1 cysts were used to produce the subsequent generation progenies. The F1 cysts however remain in a state of diapause and are not ready for hatching. They were therefore conditioned as described previously to terminate diapause^31^. A part of the activated F1 cysts was stored at 4 °C for further use, while the remaining part was hatched axenically as described above to avoid bacterial contamination. The emerged F1 larvae were further grown to maturity, after which the F2 cysts and larvae were collected. The experiment was continued until, and including, the F3 generation. During the culture period, live algae were fed *ad libitum* every day, and sterile glass bottles and sterile artificial seawater were used at all times. An aliquot of the larvae collected during each generation was subjected to bacterial challenge/resistance tests as described below. The entire procedure of pathogen challenge at F0 generation followed by offspring production and challenge assays was repeated once to confirm the reproducibility of the results.

### Survival assay of *Artemia* progenies

An aliquot of the emerged (F1 to F3) larvae collected at every generation was subjected to bacterial challenge tests as described previously^17^. Briefly, groups of 30 larvae were transferred in 7 replicates into separate sterile 40-ml glass tubes that contained 30 mL of 35 g/L sterile seawater. The larvae were challenged with *V. campbellii* or H6 strain at 10^7^ cells/mL. The survival of *Artemia* was scored at indicated time intervals.

The *Artemia* cysts from the control and treatment groups collected from all the three generations were hatched simultaneously under axenic conditions as described previously^31^ to run a common garden challenge experiment. A required number of the emerged age- and size- synchronized larvae were challenged with *V. campbellii* or H6 strain as described above. The remaining numbers were further reared under standard laboratory conditions (in triplicates) till juvenile stage (18 days old). Once at juvenile stage, animals were sampled in 3 replicates, rinsed in sterile distilled water, immediately frozen in liquid nitrogen, and stored at −80 °C for analysis of immune-related genes and epigenetic marks.

### Cyst and larvae production phenotypes

The adult *Artemia* from F1 to F3 generation of both control and treatment groups grown under common garden conditions were checked for their reproductive phenotypes i.e. production of cysts and larvae. To this end, eight mating couples from each generation and each group were transferred to Falcon tubes that contained 80 g/L seawater. The production of cysts and larvae from each couple were monitored twice a week for a period of 3 weeks after their first reproduction.

### RNA extraction and quantitative real-time PCR (RT-qPCR) analysis

Total RNA was extracted from the *Artemia* samples using the SV total RNA isolation kit (Promega, Belgium). First strand cDNA was synthesized from 1 μg total RNA using the RevertAid™ H minus First strand cDNA synthesis kit (Fermentas Gmbh, Germany) following the manufacturer’s guidelines. The expressions of *hsp70*, *hmgb1*, *Igbp*, *dscam*, *proPO, tgase*, *ftn* and *pxn* genes in the larvae were analyzed by RT-qPCR using a pair of specific primers (see supplementary Table S1). The RT-qPCR amplifications were carried out in a total volume of 25 μL, containing 9.8 μL of nuclease free water, 0.4 μL of each primer, 12.5 μL of Maxima SYBR Green qPCR Master mix (Fermentas Gmbh, Germany) and 2 μL of cDNA template. The qPCR was performed in a One Step qPCR instrument (Applied Biosystems) using a four-step amplification protocol: initial denaturation (10 min at 95 °C); 40 cycles of amplification and quantification (15 s at 95 °C, 30 s at 60 °C, and 30 s at 72 °C); melting curve (55–95 °C with a heating rate of 0.10 °C/s and a continuous fluorescence measurement) and cooling (4 °C). The *β-actin* gene was used as a reference gene for normalization of the expression of target genes. Master mixes were prepared in duplicate for each biological replicate of the sample and RT-qPCR for target and reference genes was performed. Relative quantification of target gene transcripts with a chosen reference gene transcript was done following the method described by Pfaffl *et al.* (2002)^52^ using the Relative Expression Software tool (REST^©^). These genes were selected for their highest homology against published genes but that their functional role remains to be determined e.g. by RNAi interference studies.

### Total histone extraction

Histone extraction of *Artemia* samples was carried out using the EpiSeeker histone extraction kit (ab113476; Abcam, Cambridge, UK) according to the manufacture’s instruction. Histone concentration was determined following the method of Bradford^53^.

### Analysis of histone H3 and H4 total acetylation

Histone (3 μg) samples from all the generations (F0 to F3) of the control and treatment groups were analyzed in three biological replicates for histone H3 and H4 total acetylation using the EpiSeeker histone H3 (ab131561; Abcam, Cambridge, UK) and histone H4 total acetylation detection fluorometric kits (ab131562; Abcam, Cambridge, UK), respectively, according to the manufacturer’s instructions.

### Western Blot analysis of histone H3k4me3

Histone samples were combined with loading buffer, vortexed, heated at 95 °C for 5 min, and then electrophoresed in 10% SDS-PAGE gel, with each lane receiving equivalent amounts of protein (15 μg). Heat-shocked HeLa cells (6 μg; Enzo Life Sciences, USA) were loaded in one well to serve as a positive control and for further quantification of the H3K4me3 in the samples. Gels were then transferred to polyvinylidene fluoride (PVDF) membranes (Bio-Rad, Belgium) for antibody probing. Membranes were incubated with blocking buffer [50 mL of 1X phosphate buffered saline containing 0.2% (v/v) Tween-20 and 5% (w/v) bovine serum albumin] for 60 min at room temperature and then with rabbit polyclonal anti-histone H3 (tri methyl K4) antibody (ab8580; Abcam, Cambridge, UK), which is sensitive to histone H3 trimethylation, at the recommended dilution of 1 μg/mL. Horseradish peroxidase labeled goat anti-rabbit secondary antibody (A-11034; Sigma-Aldrich, Belgium) was used as secondary antibody at the recommended dilution of 1:3000. The membranes were then treated with enhanced chemiluminescence reagent (GE healthcare, UK) and the signals were detected by a ChemiDoc MP Imaging System (Bio-Rad, Belgium). The relative signal intensity was quantified by densitometry with Bio-Rad Image Lab™ Software version 4.1.

### Statistical analysis

Survival data were subjected to logistic regression analysis using GenStat 16 (VSN International, Hemel Hempstead, UK) to determine significant differences between the control and treatment. Results for target gene expressions are presented as fold expression relative to *Artemia* actin. The expression level in the control was regarded as 1.0 and thereby the expression ratio of the treatments was expressed in relation to the control. Significant differences in the expression levels between the control and treatments were analyzed as described previously^52^. Significant differences in the epigenetic marks (histone H3 and H4 acetylation and H3K4me3) between the control and the treatment at each generation were determined by Student’s *t*-test using SPSS version 20.0 (IBM, Armonk, NY, USA). Significance level was set at P < 0.05.

## Additional Information

**How to cite this article**: Norouzitallab, P. *et al.* Probing the phenomenon of trained immunity in invertebrates during a transgenerational study, using brine shrimp *Artemia* as a model system. *Sci. Rep.* **6**, 21166; doi: 10.1038/srep21166 (2016).

## Supplementary Material

Supplementary Information
