# Supplementary material for: Probing the phenomenon of trained immunity in invertebrates during a transgenerational study, using brine shrimp Artemia as a model system
Source: Sci Rep. 2016 Feb 15;6:21166. doi: 10.1038/srep21166 (PMC4753410; doi:10.1038/srep21166)
Supplement: Supplementary Information [file srep21166-s1.pdf]

Supplementary information:

Probing the phenomenon of trained immunity in invertebrates during a transgenerational study, using brine shrimp *Artemia* as a model system

Parisa Norouzitallab[1,2], Kartik Baruah[1], Priyanka Biswas[1], Daisy Vanrompay[2] & Peter Bossier[1]

[1]Laboratory of Aquaculture & *Artemia* Reference Center, Department of Animal Production, Faculty of Bioscience Engineering, Ghent University, Rozier 44, Ghent 9000, Belgium, [2]Lab of Immunology and Animal Biotechnology, Faculty of Bioscience Engineering, Ghent University, Coupure 653, Ghent 9000, Belgium.

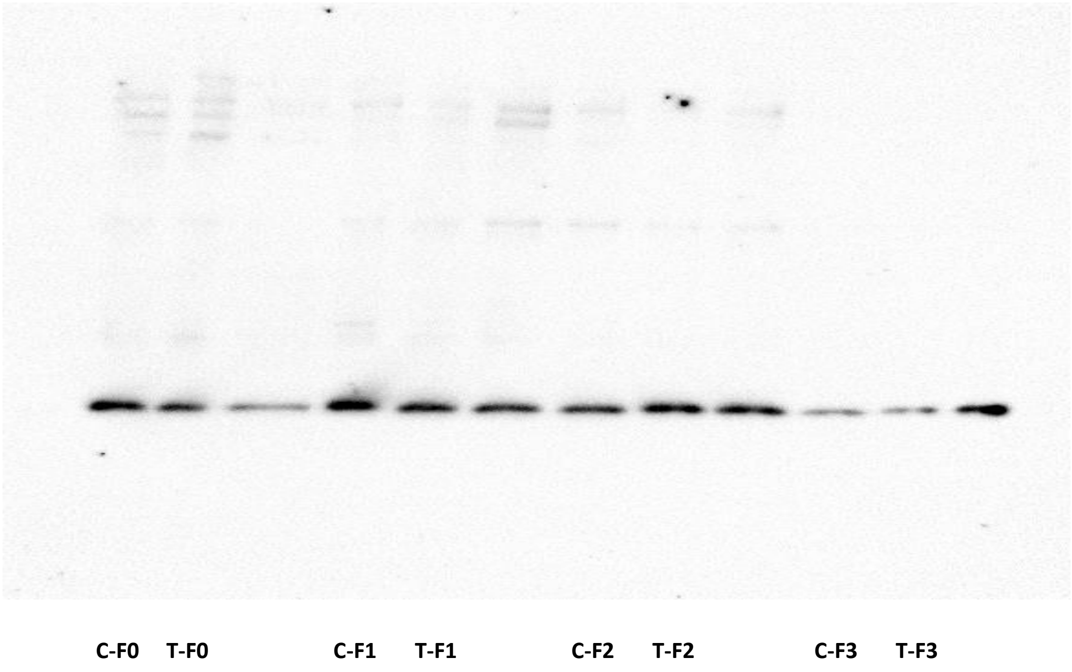

Figure S1 | Uncropped immunoblot image showing that *Vibrio campbellii*-exposed parental generation and their unexposed progenies exhibited stochastic pattern in the trimethylation levels of histone H3 at lysine 4 tail (H3K4me3). See Figure 9 for explanation of the experimental groups. Total histone extracted from F0 to F3 juveniles, reared under common garden conditions, was resolved in SDS-PAGE gel, transferred to polyvinylidene fluoride membrane and then probed with anti-H3K4meE primary antibody. 15 µg of *Artemia* histone protein was loaded in each lane. HeLa cells (6 µg) were loaded onto

one well to serve as a positive control and for calculating the amount of histone H3K4me3 in the sample.

Table S1 | Specific primers used for RT-qPCR analysis of *Artemia* heat shock protein 70 (*hsp70*), high mobility group box 1 protein (*hmgb1*), prophenoloxidase (*proPO*), transglutaminase (*tgase*), ferritin (*ftn*), peroxinectin (*pxn*) and *β-actin*. In each pair, forward primers are presented first followed by the reverse primer.

Gene	Sequences of forward and reverse primers (5'-3')
hsp70	cgataaaggccgtctctcca
	cagcttcaggtaacttgtccttg
hmgb1	ggatgaaagcaaaccccgtg
	gtgctcttctctgcaagtctg
proPO	tctgcaaggaggatttaagga
	tgactgacaaaggagatgggac
tgase	tctctccgtgtctctccaaaag
	ccccacaagaagcatctgaag
ftn	tccaaggcttatccgatgaaca
	atgaccaagtgagtgcttctct
pxn	gagctaccgatgaagatccag
	cgtttcctgaacagcgaataaa
β-actin	agcggttgccatttcttgtt
	ggtcgtgacttgacggactatct
